# Metabolic perturbations in cardiomyopathies: implications for early diagnosis and targeted interventions

**DOI:** 10.3389/fcvm.2025.1616677

**Published:** 2025-10-17

**Authors:** Rula Al-Shahrabi, Ghadeera Al Mansoori, Muna Al-Saffar, Nadia Akawi

**Affiliations:** ^1^ Sharjah Institute for Medical and Health Sciences, University of Sharjah, Sharjah, United Arab Emirates; ^2^ Department of Cardiology, Sheikh Shakhbout Medical City, Abu Dhabi, United Arab Emirates; ^3^ Department of Genetics and Genomics, College of Medicine and Health Sciences, United Arab Emirates University, Al Ain, United Arab Emirates; ^4^ Division of Genetics and Genomics, Boston Children’s Hospital, Boston, MA, United States; ^5^ Division of Cardiovascular Medicine, University of Oxford, Oxford, United Kingdom

**Keywords:** metabolomics, cardiomyopathy, LC-MS, cardiovascular disease, heart failure

## Abstract

Cardiomyopathy (CM) is a heterogeneous group of diseases characterized by structural and functional changes in the heart, with the exact cause often remaining unknown. CM can arise from both inherited and acquired metabolic disturbances. Alterations in energy production and substrate utilization impair the heart's contractile function and limit its ability to respond to stress. Given the complexity and dynamic nature of CM, as well as the multiple etiologies involved, we reviewed metabolomic studies employing high-throughput platforms to understand how metabolic pathways shift across CM subtypes and how these perturbations may inform clinical translation. Several recurring disruptions emerge across CM with alterations in amino acid metabolism (valine, leucine, methionine, tryptophan, tyrosine); mitochondrial redox imbalance (NAD/NADH shifts, niacinamide, acylcarnitines); and oxidative stress as central hallmarks. Each subtype, however, displays a different emphasis. For instance, hypertrophic CM is characterized by nucleotide remodeling, particularly in cases involving *MYBPC3* mutations; dilated CM shows accumulation of Krebs cycle intermediates and trimethylamine-N-oxide; restrictive CM is associated with amino acid stress related to amyloidosis; tachycardia-induced CM involves fatty acid remodeling and elevated uric acid, while Takotsubo CM is linked to ketone utilization and glutamate excitotoxicity. Overall, a single metabolomic profile cannot capture CM. What emerges from this review is that subtype-specific shifts, and the way they interact, provide meaningful insight into disease mechanisms and highlight pathways with diagnostic, prognostic, and therapeutic relevance. This broader perspective shifts the focus beyond narrow comparisons, making the translational relevance of metabolomics in CM more apparent.

## Introduction

The World Heart Report 2023 identifies cardiovascular diseases (CVD) as the leading global cause of mortality, accounting for over 20.5 million deaths in 2021 and representing a significant burden of premature non-communicable disease deaths (1, 2). Cardiomyopathies (CM), a subset of CVD, are defined by structural and functional myocardial changes such as altered size, shape, or wall thickness arising from either ischemic (coronary artery disease-related) or non-ischemic origins (3, 4). The Centers for Disease Control and Prevention (CDC) notes that CM etiology often remains idiopathic. However, it may be inherited or acquired, with risk factors including hypertension, arrhythmias, valvular diseases, endocarditis, and metabolic disorders such as diabetes mellitus, Gaucher disease, and mitochondrial dysfunction (5, 6).

CM subtypes, hypertrophic (HCM), dilated (DCM), arrhythmogenic (ACM), restrictive (RCM), left ventricular noncompaction, and Takotsubo, exhibit distinct morphological, genetic, and metabolic profiles (7). In healthy adult hearts, fatty acid oxidation generates approximately 60% of adenosine triphosphate (ATP), with carbohydrates as a secondary substrate. In CM, heightened energy demands drive substrate flexibility, utilizing glucose, fatty acids, and ketone bodies. Disruptions in ATP production rates and substrate preference impair contractility and stress responses, hallmarks observed across CM subtypes (8–11).

**Figure 1:**
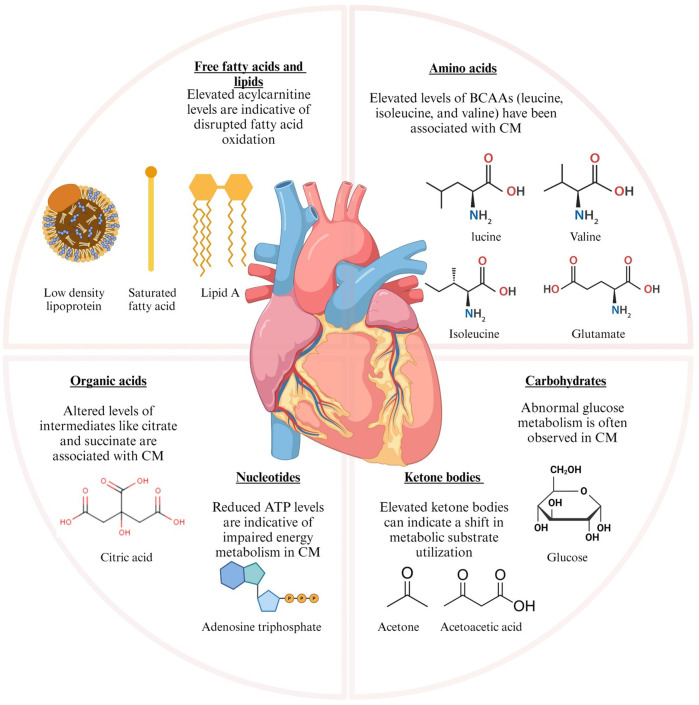
Metabolic alterations in cardiomyopathy and cardiovascular diseases leading to metabolic shifts. The graphic illustrates the principal metabolic alterations identified in cardiomyopathy (CM) and ischemic heart failure. The metabolic changes are classified into various categories based on their biological activities. BCAAs, branched-chain amino acids; CM, cardiomyopathy; ATP, adenosine triphosphate; LDL, low-density lipoprotein. Figure created with BioRender.com.

Metabolomics, the systematic study of metabolites (amino acids, fatty acids, carbohydrates, nucleotides), provides a window into these biochemical shifts. Key metabolites linked to CM, including branched-chain amino acids (BCAAs), acylcarnitines, glucose, and ketone bodies, reflect altered pathways such as glycolysis and lipid metabolism, offering potential diagnostic and therapeutic targets (Figure 1) (12, 13). These metabolites demonstrate the metabolic pathway changes that occur in CM (14–16), highlighting the disease mechanisms and acting as potential biomarkers for diagnosis and treatment. Although the genetic and structural features of CMs are well established, the metabolic dimension has not been systematically addressed. Previous studies have often restricted their focus to narrow pairwise comparisons, which has left a fragmented view of the field. In this review, we seek to close that gap by bringing together evidence on metabolic remodeling across the full spectrum of CM phenotypes. We aim to move beyond earlier fragmented investigations and to highlight the translational promise of metabolomics for enhancing diagnosis, refining risk stratification, and informing therapeutic strategies in CM.

## Cardiomyopathy classifications

**Figure 2:**
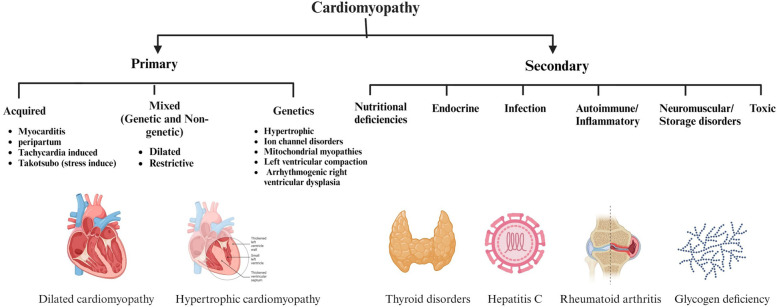
Classification and causes of cardiomyopathy (CM) based on the American Heart Association. This figure categorizes CM into primary and secondary forms, emphasizes the variety of etiologies that can cause CM, including acquired and genetic manifestations, as well as a range of systemic and metabolic conditions. Figure created with BioRender.com.

The American Heart Association classifies CM into primary (genetic, acquired, or mixed) and secondary (systemic/non-cardiac) forms (Figure 2) (17).

## Primary cardiomyopathy

HCM, the most common genetic CM, involves left ventricular wall thickening, often due to autosomal dominant mutations in genes such as *MYH7*, *MYBPC3*, or *TNNT2* (18). Arrhythmogenic right ventricular cardiomyopathy (ARVCM), another genetic form, features fibrofatty tissue infiltration in the right ventricle, causing arrhythmias. About 66% of patients test positive for causative genetic mutations (19–21). This tissue replacement impairs contraction, reduces oxygen utilization, and lowers ATP production, thereby driving metabolic shifts and the accumulation of diacylglycerols and ceramides (22, 23).

Acquired CM result from external factors, such as infections in viral myocarditis, pregnancy in peripartum CM, or stress in Takotsubo CM (20). In Takotsubo, stress-induced catecholamine release causes myocyte injury, impairs perfusion, dysregulates fatty acid metabolism, increases inflammatory metabolites (chemokines, cytokines), and elevates oxidative stress via reactive oxygen species (ROS) (24, 25). Mixed CM is a combination of genetic and acquired types associated with both dilated and restrictive forms. DCM involves left ventricle dilation and impaired contraction (17), driven by acquired factors such as myocarditis, cardiotoxins such as alcohol, antineoplastic agents, and metabolic disorders, including insulin resistance, obesity, dyslipidemia, and diabetes. These conditions alter energy utilization, increase oxidative stress, and promote inflammation and fibrosis, often progressing to heart failure (26–28).

RCM features nondilated ventricles with impaired diastolic function, causing wall stiffening and restricted filling (29). Amyloidosis, a primary cause of RCM, involves amyloid protein deposits in the myocardium, leading to cardiomyocyte separation, toxicity, and stiffness (30). These deposits disrupt lipid metabolism, increase lipid peroxidation, reduce mitochondrial respiration, and contribute to severe heart failure (29, 30).

## Secondary cardiomyopathy

Endocrine disorders, such as diabetes, thyroid disorders, and obesity, significantly alter carbohydrate and lipid metabolism, frequently increasing ketone body levels (17, 31–33). Viral infections, on the other hand, increase anaerobic glycolysis and hypoxia, resulting in high levels of ROS and lactate (34). Glycogen storage disorders lead to muscular dystrophies and further dysregulate ATP and creatine phosphate levels (35). Nutritional deficiencies, including vitamin C, selenium, and thiamine, disrupt carbohydrate metabolism, leading to elevated pyruvate and lactate levels (36). Toxic causes such as alcohol, anabolic steroids, and radiation disrupt various metabolic pathways through the production of acetaldehyde (37). Collectively, these metabolites play significant roles in the pathogenesis of secondary CM (6).

## Metabolomic profiling of cardiovascular diseases

The Human Metabolome Project (HMDB), launched two decades ago, has enabled the quantification of metabolites in body fluids, aiding biomarker discovery, early diagnosis, disease progression, therapeutic response, and drug development (38). Key techniques include NMR and MS platforms, such as liquid chromatography-tandem mass spectrometry (LC-MS/MS) or gas chromatography-tandem mass spectrometry (GC-MS) (39). NMR facilitates metabolite identification with minimal sample preparation, while MS offers high sensitivity for ionized molecules (40). High-resolution ¹H NMR provides reproducible results and fast data acquisition (9, 39, 40). Carbon NMR (^13^C-NMR) reveals structural details, identifies functional groups, and provides more comprehensive positional information compared to ^1^H-NMR (39). GC-MS requires derivatization for volatile metabolites (41). Whereas LC-MS enables analysis of both polar/non-polar metabolites in complex samples such as blood or urine with minimal derivatization (42).

A single metabolomic approach cannot fully capture the metabolome. Psychogios et al. integrated ^1^H-NMR, GC-MS, and LC-MS in heart transplant patients, identifying key metabolites like glucose and lactic acid (indicating anaerobic glycolysis in ischemic CM) and arachidonic acid in atherosclerosis (43). High acylcarnitine ratios (C4/C18:2) signal mitochondrial dysfunction and increased CAD risk in diabetic patients (44). Anlar et al. found altered carbohydrate, steroid, and fatty acid metabolism in CAD using UPLC-MS, with elevated mannitol, glucose, and oleoylcarnitine linked to CAD risk (45). Fu et al. noted phospholipid and fatty acid changes such as lysoPC (18:2), lysoPE (20:3), and MG (20:0) aiding CAD diagnosis (46). Harshfield et al. identified creatine and sphingomyelin (d18:2/24:1) associated with cerebral small vessel disease severity using UPLC-MS and NMR from different ethnicities (Caucasians, Caribbean, and African) (47). In peripheral artery disease (PAD), altered lipoprotein and phospholipid metabolism were shown to correlate with mortality (48), while phenylalanine, tyrosine, and oxidized low-density lipoprotein (oxLDL) were associated with arterial stiffness (49). Ismaeel et al. used LC-MS to show phenylalanine/tyrosine ratios and ceramides indicating PAD severity (50).

Rheumatic heart disease (RHD) is characterized by cumulative valvular lesions resulting from acute rheumatic fever and often remains undiagnosed until reaching an advanced stage (51). Das et al. revealed significantly altered pathways, particularly in D-glutamine, D-glutamate, and linoleic acid metabolism via untargeted LC-MS analysis (52). Furthermore, N-acetylneuraminate and arachidonic acid were identified as the primary metabolites, considering them promising therapeutic biomarkers. Congenital heart disease depends on fetal echocardiography. Despite this examination, some cases might be missed. Untargeted metabolomics can detect low-concentration metabolites and predict different subtypes (53). Dong et al. detected changes in nicotinamide adenine dinucleotide (NAD), glutamine, and arginine levels, indicating hypoxia-driven metabolic remodeling (54).

In deep venous thrombosis, overdiagnosis or underdiagnosis due to the poor specificity and moderate sensitivity of D-dimer testing and imaging can lead to fatal pulmonary embolism or increased bleeding risk from excessive anticoagulant use (55). Altered carnitine, glucose, and tryptophan metabolism dysregulate glycolysis and lipid pathways, aiding early diagnosis (56). Chen et al. noted tryptophan derivative DT-26 as an antithrombotic (57). Culler et al*.* linked myoinositol and carnitine to subclinical cardiac dysfunction in heart failure with preserved ejection fraction (HFpEF) using ^1^H-NMR (58).

However, the reviewed studies exhibit critical limitations that significantly impair their clinical impact. Small and potentially non-representative sample sizes, a lack of mechanistic depth, reliance on non-specific markers, the absence of diagnostic metrics, limited focus on clinical translation, and the use of cross-sectional designs collectively restrict their generalizability and applicability. Addressing these shortcomings through larger, more diverse cohorts, in-depth mechanistic studies, quantitative validation, longitudinal designs, and practical implementation strategies will be essential to enhance their utility in advancing precision medicine for cardiovascular disease.

## Metabolic signature in primary cardiomyopathy

### Hypertrophic cardiomyopathy

HCM, a genetic disorder caused by mutations in various genes, poses diagnostic challenges due to variable gene expression within families and phenotypic overlap with conditions like amyloidosis and hypertensive heart disease, risking misdiagnosis (59, 60). Often undetected until complications like sudden cardiac death or heart failure arise, HCM is typically diagnosed using cardiovascular magnetic resonance imaging and electrocardiograms (60). Emerging metabolomic studies offer more profound insights (Table 1). Wang et al. used targeted LC-MS/MS and lipidomics to identify dysregulated metabolites in glycolysis, the Krebs cycle, purine/pyrimidine metabolism, and carnitine synthesis in HCM tissues and plasma, and noted significant alterations in the pentose phosphate pathway and oxidative stress (61). They identified three metabolic subgroups: Subtype 1, characterized by elevated Krebs cycle metabolites such as isocitrate, was associated with better survival, whereas Subtype 2, marked by high purine/pyrimidine metabolites and short-chain carnitines, was linked to worse outcomes. Together, these profiles suggest that such metabolites may serve as both diagnostic and prognostic tools.

**Table 1:** Representative metabolite alterations in HCM with clinical relevance.

Metabolites	Clinical outcomes	Translational implications
Dimethylglycine, N-acetyl-L-glutamine, β-aminobutyric acid, XMP, GMP, PC38:6p (16:0/22:6), TAG52:2 (C18:0)	Associated with adverse prognosis; predictive of overall survival	Serve as biomarkers of disease severity and mortality risk
PG38:6 (18:2/20:4), UDP-galactose, branched-chain amino acids, NADP, acetyl-CoA, NADH	Reflect increased oxidative stress, inflammatory activation, and impaired mitochondrial respiration	Highlight potential druggable pathways for therapeutic modulation in HCM
PE32:0 (16:0/16:0), phosphocreatine, 1,3-dimethyluracil, uric acid	Linked to impaired myocardial contractility; metabolic signature associated with MYBPC3 mutations	Useful as diagnostic/prognostic biomarkers and as genotype-specific metabolic readouts

Key metabolites identified in the hypertrophic cardiomyopathy subtype. These metabolites have been linked to diagnostic accuracy, treatment monitoring, risk stratification, and potential therapeutic targeting, underscoring their value for advancing clinical practice.

Previs et al., using LC-MS-based proteomics and targeted metabolomics, reported reduced long-chain acylcarnitine, a shift to ketone metabolism (elevated 3-hydroxybutyrate), and increased branched-chain amino acids in HCM, reflecting reduced ATP availability and impaired contractility (62). In HCM patients with *MYBPC3* variants, alterations in acylcarnitine, histidine, lysine, purine, and steroid hormones were associated with disease progression (63). Comparing Dutch *MYBPC3* carriers with severe vs. mild/no symptoms, the study identified potential biomarkers for disease severity, offering insights into mechanisms and targeted therapies. Current metabolomic studies in HCM face significant hurdles that limit their translational potential. Uncontrolled external factors like diet, medication (e.g., beta-blockers), and comorbidities introduce confounding variables, while variability in control samples (age, sex differences) undermines biomarker specificity. The predominant focus on tissue-level changes, rather than systemic biomarkers, restricts non-invasive clinical use, and the emphasis on *MYBPC3* variant carriers or end-stage HCM patients limits generalizability across genotypes and disease stages. There is growing evidence that links impaired mitochondrial metabolism arising from mutations in either mitochondrial or nuclear DNA to the development of CM, with recent investigations emphasizing their role in disease mechanisms and as potential therapeutic targets (64). Nollet and colleagues, for instance, demonstrated that mitochondrial dysfunction in HCM disrupts cardiomyocyte structure. Yet, these defects could be reversed with the cardiolipin-stabilizing compound elamipretide, which enhances NAD^+^ availability and supports respiration. Their findings highlight the potential of mitochondria-targeted therapies, particularly in genotype-negative HCM patients (65). In a complementary line of work, Franco and collaborators identified a rare *MFN2* mutation (R400Q) that selectively impairs mitophagy, leading to a newly recognized entity termed “mitophagy CM” (66). This mutation, identified in both HCM and DCM and found more frequently among individuals of African ancestry, points to ancestry-specific risks and underscores the value of genetic screening for mitophagy-related defects in otherwise unexplained cases (66).

To enhance translation, future research should control for external factors, match control groups, prioritize blood/urine biomarkers, and include diverse HCM genotypes and stages for broader applicability in early diagnosis and personalized treatment.

### Arrhythmogenic cardiomyopathy

Arrhythmogenic CM is a genetically complex disease with variable severity and clinical presentation, which complicates diagnosis even with traditional methods. Metabolomic profiling offers a comprehensive approach to screening and characterization in ACM (67, 68). Volani et al. identified key metabolites in ACM patients’ plasma, revealing dysregulated pathways (68). Notably, *α*-Aminoadipic acid was found to be downregulated, indicating disrupted lysine degradation. At the same time, propionyl carnitine and asymmetric dimethylarginine were upregulated, pointing to endothelial dysfunction via altered beta-oxidation and nitric oxide synthesis pathways. These metabolites highlight potential biomarkers and therapeutic targets for ACM management.

### Dilated cardiomyopathy

DCM, a leading cause of heart failure in adults, presents challenges due to its complex pathophysiology, including systolic dysfunction, ventricular dilation, connective tissue thickening, heart enlargement, and metabolic alterations (28). Standard diagnostic biomarkers like B-type Natriuretic Peptide (BNP), NT-proBNP, troponins, and galectin-3, alongside clinical evaluation and echocardiogram imaging, often lack sensitivity and accuracy to fully capture DCM's complexity and prognosis, especially in comorbid patients (69). Targeted metabolomics offers broader molecular coverage, making it a valuable tool for studying complex diseases (70).

Ampong reviewed metabolomic profiles of DCM in serum, plasma, and heart tissues using GC-MS, LC-MS, UHPLC, and NMR (Table 2) (71). Upregulated metabolites included methylhistidine (protein degradation), prolylhydroxyproline (cardiac remodeling), and isoleucine, linoleic acid, BCAA, and phenylalanine (dysregulated amino acid metabolism, oxidative stress). Other elevated metabolites were glucose, serine, acetate, dimethylsulfone, hypoxanthine, creatinine, creatine, and trimethylamine-N-oxide (TMAO) compared to ischemic CM. Glutamine, piperine, and citrulline were also increased, reflecting metabolic adaptation to stress. Downregulated metabolites included threonine (impaired protein synthesis), histidine, tryptophan, lactate, methionine, formate, arginine, and hydroxytetradecanoylcarnitine. Dysregulation of amino acid and lipid metabolism offers potential biomarkers for differentiating DCM from ischemic CM and for monitoring disease progression.

**Table 2:** Representative metabolite alterations in DCM with clinical relevance.

Metabolites	Clinical outcomes	Translational implications
Acylcarnitines, succinic acid, malate, methylhistidine, aspartate, methionine, phenylalanine	Associated with the presence of DCM	Support early diagnosis
Acylcarnitines, isoleucine, linoleic acid, tryptophan	Reflect changes with treatment	Useful for monitoring therapy response
1-pyrroline-2-carboxylate, lysophosphatidylinositol (16:0/0:0), phosphatidylglycerol, norvaline,phosphatidylcholine	Show distinct metabolic patterns in DCM vs. ICM	Aid in non-invasive differential diagnosis
Trimethylamine-N-oxide, creatine, creatinine, lactate	Linked with prognosis in HF secondary to DCM	Predict risk of mortality and guide risk stratification

Key metabolites identified in the dilated cardiomyopathy subtype. These metabolites have been linked to diagnostic accuracy, treatment monitoring, risk stratification, and potential therapeutic targeting, underscoring their value for advancing clinical practice.

Verdonschot et al. performed targeted metabolomics on plasma and urine from DCM patients, using NT-proBNP as a reference (27). The High levels of C16-acylcarnitine, glutamic acid, sialic acid, and cystathionine in severe DCM indicated dysregulated beta-oxidation, mitochondrial dysfunction, systemic inflammation, and oxidative stress, all strongly correlated with NT-proBNP. Conversely, low levels of 3-methylhistidine, urine carnosine, 3-hydroxyisovaleric acid, and citric acid showed an inverse correlation, suggesting reduced protective metabolic responses. These findings support the use of acylcarnitine and amino acid metabolites as adjuncts to NT-proBNP for improved severity stratification in DCM.

Vignoli et al., using untargeted 1H-NMR on serum, confirmed similar disruption in energy and lipid metabolism, inflammation, oxidative stress, and gut microbiome interactions in DCM-induced heart failure (72). High creatinine, succinic acid, lactate, LDL-triglycerides, and TMAO were linked to higher mortality risk, while lower creatine, ApoA2, and HDL phospholipids indicated better outcomes. Combining metabolomics with left ventricular ejection fraction improved prognostic accuracy (hazard ratio: 7.47 to 8.09) compared to NT-proBNP alone. The clinical implications demonstrate the predictive value of serum metabolic markers in predicting mortality and enhancing risk stratification beyond NT-proBNP.

Zhao et al. analyzed plasma metabolomics in DCM and Ischemic CM heart failure patients compared with healthy controls using untargeted LC-MS/MS (73). In DCM, reduced 1-pyrroline-2-carboxylate and altered norvaline levels suggested impaired oxidative stress response and anti-inflammatory capacity, with disruptions in alpha-linolenic acid metabolism. In Ischemic CM, decreased lysophosphatidylinositol (16:0/0:0) reflected altered adrenergic signaling, while elevated phosphatidylglycerol (6:0/8:0) and fatty acid esters pointed to mitochondrial and lipid metabolism dysregulation. Pathways like linoleic acid metabolism, arginine biosynthesis, and D-glutamine/D-glutamate metabolism were dysregulated in Ischemic CM. Both conditions showed altered phosphatidylcholine (18:0/18:3) and glycerophospholipid metabolism, highlighting shared metabolic disturbances. The reviewed studies shed light on the metabolic complexity of DCM, highlighting the potential of plasma-based metabolomics in enhancing diagnostic accuracy and patient stratification.

Overall, these studies highlight the metabolic complexity of DCM and the utility of plasma and tissue-based metabolomics for diagnosis, prognosis, and patient stratification. Vignoli et al. identified a metabolic signature predictive of mortality, Zhao et al*.* highlighted clear metabolic distinctions between DCM and ischemic CM, and Ampong further revealed disrupted lipid and amino acid pathways central to disease progression. However, all studies remain limited by small sample sizes, methodological variability, and cross-sectional designs, underscoring the need for larger, longitudinal studies with standardized protocols to harness the clinical potential of metabolomics in DCM fully.

### Restrictive cardiomyopathy

Cardiac Amyloidosis **(**CA) is a well-established cause of RCM, yet few studies have characterized its metabolic signature (74). In CA, deposition of immunoglobulin light chain (AL) and transthyretin (ATTR) amyloid leads to ventricular thickening and impaired filling. Diagnosis is challenging and often requires advanced modalities, such as cardiac magnetic resonance, positron emission tomography, or endomyocardial biopsy (74). Proteomic analysis using Nano LC-MS/MS has successfully differentiated CA subtypes (AL, ATTR, serum amyloid A) by detecting amyloid signature proteins like ApoE, ApoA-IV, and SAP, which were upregulated in affected tissues. At the same time, conventional cardiac markers such as troponins I and T were decreased (75).

These shifts indicate misfolded protein accumulation and associated cardiac dysfunction in RCM and suggest that proteomic and metabolomic profiling can differentiate CA subtypes and may complement or reduce reliance on invasive biopsy.

Additionally, Olsson et al. applied untargeted metabolomics (GC-MS and LC-MS) to plasma from ATTRV30M patients, identifying reduced tryptophan, phenylalanine, and tyrosine, along with altered malic acid and acylcarnitines (Table 3). Alterations in plasma amino acids and acylcarnitines may serve as non-invasive biomarkers for monitoring disease progression in ATTR amyloidosis (76).

**Table 3:** Representative metabolite alterations in RCM with clinical relevance.

Metabolites	Clinical outcomes	Translational implications
Tryptophan, phenylalanine, tyrosine, maltose	Indicators of oxidative stress, endothelial dysfunction, and adrenergic stress	Early biomarkers for diagnosis and stratification, especially in amyloidosis-related RCM
Leucine, ketoleucine, malic acid, valine	Reflect mitochondrial dysfunction and impaired contractile performance	Prognostic markers for mitochondrial impairment; aid in detecting restrictive changes before overt heart failure
Leucine, niacinamide	Capture metabolic adaptation to energy stress	Candidates for therapeutic monitoring, particularly in NAD⁺ augmentation strategies

Key metabolites identified in the restrictive cardiomyopathy subtype. These metabolites have been linked to diagnostic accuracy, treatment monitoring, risk stratification, and potential therapeutic targeting, underscoring their value for advancing clinical practice.

### Inflammatory cardiomyopathy

Myocarditis, an inflammatory CM, is often caused by viruses such as Coxsackie A/B, leading to cardiac dysfunction and remodeling (77, 78). Diagnosis remains difficult due to the non-specificity of biomarkers like troponins and C-reactive protein, and the limited sensitivity of imaging and invasive endomyocardial biopsy (EMB) (79). Although emerging biomarkers such as microRNAs show potential, they are not yet clinically established. Metabolomic profiling offers rapid detection of metabolic shifts, with notable dysregulation in lipid metabolism and amino acid biosynthesis. The PGC-1α/PPARγ axis, central to energy metabolism, appears to be particularly affected (80).

Kong et al. used NMR-based metabolomics in CVB3-induced myocarditis mouse models, revealing elevated leucine, isoleucine, valine, lysine, and 3-hydroxybutyrate in chronic myocarditis, and reduced alanine, glutamate, lactate, and succinate in both acute and DCM stages (81). Disrupted pathways included nicotinamide, alanine-glutamate, and taurine metabolism. The present results may help differentiate between acute, chronic, and dilated cardiomyopathy–transition stages of myocarditis.

A complementary UPLC-MS/MS study by Kong et al. found elevated metabolites such as betaine, shikimic acid, and estrone 3-sulfate, which correlated with gut microbes such as Streptococcus and Enterococcus, linking gut dysbiosis to myocarditis-induced metabolic disturbances (82). Integration of metabolomics with microbiome profiling may provide novel approaches for early diagnosis and therapeutic targeting in viral myocarditis.

### Tachycardia-induced cardiomyopathy

Tachycardia-induced CM (TICM) is a reversible form of CM linked to persistent arrhythmias, particularly atrial fibrillation (AF) and atrial flutter. Still, in severe cases, it can progress to cardiogenic shock or sudden death (83, 84). Standard diagnostics include ECG, echocardiography, cardiac MRI, and plasma BNP/NT-proBNP levels, yet these markers lack specificity, making early differentiation from other cardiomyopathies challenging (85).

Lu et al. developed a metabolomics-based model using non-targeted GC-MS and LC-MS to analyze serum from various AF subtypes, identifying increased D-glutamic acid, uric acid, and 2-ketoglutaric acid, and decreased oleic acid and glycerol-2-phosphate across all AF types (86). Unique metabolite changes were observed in AF-related stroke, indicating disruptions in glutamate, glycerophospholipid, and fatty acid metabolism, which may contribute to arrhythmogenesis by promoting membrane instability and energy dysregulation. The current observations could improve early AF/TICM diagnosis and identify high-risk patients predisposed to stroke.

Tu et al. further explored metabolic rewiring in TICM using hiPSC-derived cardiac tissues and a canine tachypacing model (87). They observed increased glucose metabolites (pyruvate, sorbitol, succinic acid) and reduced expression of genes involved in oxidative phosphorylation and fatty acid oxidation (Table 4). This metabolic shift toward glycolysis impaired NAD+ redox balance and increased protein acetylation, including that of SERCA2a, leading to contractile dysfunction. These findings underscore the role of altered energy metabolism in TICM pathogenesis and its potential as a therapeutic target.

**Table 4:** Representative metabolite alterations in TICM with clinical relevance.

Metabolites	Clinical outcomes	Translational implications
Oleic acid, D-glutamic acid, uric acid, L-acetylcarnitine, decanoylcarnitine, stearic acid, creatinine	Linked with recurrence, oxidative stress, and structural remodeling	Can function as diagnostic markers for recurrence and patient stratification
L-lysine, valine, tyrosine, methionine, alanine; evidence of glycolytic shift, protein acetylation, and NAD redox imbalance	Associated with impaired contraction and mitochondrial dysfunction	Useful for therapeutic assessment, particularly when evaluating metabolic modulators

Key metabolites identified in the tachycardia induced cardiomyopathy subtype. These metabolites have been linked to diagnostic accuracy, treatment monitoring, risk stratification, and potential therapeutic targeting, underscoring their value for advancing clinical practice.

### Takotsubo/stress cardiomyopathy

Takotsubo Syndrome (TTS) or stress CM is often misdiagnosed as myocardial infarction (MI) due to overlapping features like ECG abnormalities and elevated BNP/NT-proBNP levels. However, these biomarkers lack specificity for TTS and can also be elevated in cardiac and systemic conditions (88, 89). Despite evidence of myocardial inflammation and oxidative stress in TTS, systemic biochemical validation remains limited.

Vanni et al. used ¹H-NMR metabolomics to analyze serum from TTS patients, revealing increased ketone bodies (2-hydroxybutyrate, acetyl-L-carnitine, glutamate) and decreased amino acid metabolites (Table 5) (88). These findings indicate systemic oxidative stress and metabolic dysregulation, which correlated with reduced left ventricular ejection fraction (LVEF), suggesting potential diagnostic and prognostic value.

**Table 5:** Representative metabolite alterations in Takotsubo CM with clinical relevance.

Metabolites	Clinical outcomes	Translational implications
Ketone bodies, 2-hydroxybutyrate, glutamate	Higher ketone levels track with disease severity (LVEF decline, arrhythmia risk, ventricular remodeling), redox imbalance reflects stress response; glutamate excess linked to excitotoxicity and poor contractile reserve	Prognostic markers of severity, point to oxidative stress as a treatment target, help in patient risk stratification
Acetyl-L-carnitine	Signals mitochondrial dysfunction	Can be followed as a therapeutic monitoring marker, indicating recovery of mitochondrial function
Reduced amino acids	Indicate catabolic stress and are associated with myocardial injury and remodeling	Provide rationale for supportive metabolic strategies, including amino acid supplementation

Key metabolites identified in the Takotsubo cardiomyopathy subtype. These metabolites have been linked to diagnostic accuracy, treatment monitoring, risk stratification, and potential therapeutic targeting, underscoring their value for advancing clinical practice.

Scally et al. observed that TTS patients, even with preserved LVEF, exhibited reduced peak VO₂ and elevated VE/VCO_2_ slope, consistent with a heart failure-like phenotype and raising concerns about long-term effects on cardiac function (90). Functional capacity testing may therefore be valuable for detecting persistent cardiac impairment in TTS patients, even after apparent recovery.

Further, a study by Chou et al. using a mouse model of isoprenaline-induced stress demonstrated sustained cardiac damage due to a metabolic shift toward anabolic glucose pathways (glycolysis, hexosamine biosynthesis), persistent oxidative stress, and progressive structural remodeling (91). These findings underscore the importance of early intervention targeting metabolic alterations to prevent long-term cardiac consequences in TTS.

### Metabolic signatures in secondary cardiomyopathy

**Figure 3:**
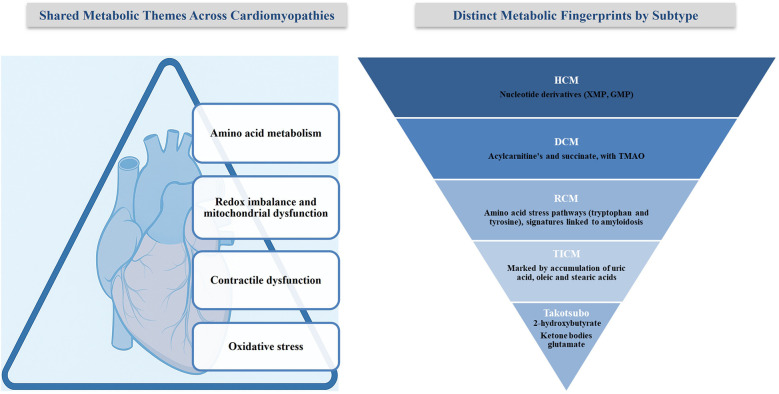
Shared and distinct metabolic alterations in cardiomyopathies. The left panel summarizes shared metabolic themes across CM subtypes. The right panel highlights distinct metabolic fingerprints unique to each subtype.

Secondary CM are heart conditions caused by a mix of factors such as tropical living environments, poverty, poor nutrition, and limited access to healthcare. Genetic predisposition also influences how individuals respond to infections. This review highlights how metabolic disturbances such as deficiencies in thiamine, selenium, and calcium, as well as immune-mediated alterations or toxin exposure, contribute to the development of these conditions (Table 6, Figure 3). It also discusses how these insights may inform targeted treatment strategies (92).

**Table 6:** Overview of metabolic signatures in human models of primary and secondary cardiomyopathy.

CM subtype	Sample type	Cohort	Ethnicity	Metabolic high throughput technique/methods	Upregulated metabolites	Downregulated metabolites	Dysregulated pathways	Ref
Hypertrophic Cardiomyopathy (HCM)	Cardiac tissue Plasma	Cardiac tissues: 349 patients (with HCM) and 16 controls (without HCM) Plasma: 143 patients (with HCM), 60 controls (without HCM) and 46 patients (with DCM)	Asian (Chinese)	Targeted metabolomics, lipidomics and proteomics analysis.	Purine and pyrimidine nucleotides, Short-chain carnitines	Long-chain carnitines, S-adenosylmethionine	Vit B6 metabolism, Pentose phosphate pathway, Purine and Pyrimidine Metabolism, Glutathione Metabolism	(61)
	Cardiac tissue	29 HCM patients carrying variants in *MYBPC3* and *MYH7*, 10 HCM patients with no sarcomere variants, 8 individuals with no significant heart disease as a control group	Caucasian	Proteomics and targeted metabolomics.	Ketone bodies, lactate	Long-chain acylcarnitine's ATP Phosphocreatine NADH NADPH Acetyl-CoA	Fatty Acid Oxidation Ketone Body Metabolism Amino Acid Metabolism Nucleotide Metabolism	(62)
	Plasma sample	30 HCM patients carriers of *MYBPC3* variants with sever phenotype 30 HCM patients carriers of *MYBPC3* with no/mild phenotype 10 HCM patients with no variants in *MYBPC3*	European	Untargeted and targeted Metabolomics using direct-infusion high-resolution mass spectrometry	Acylcarnitine Histidine Lysine	Di- and oligopeptides Steroid hormone metabolites	Acylcarnitine Metabolism Histidine Metabolism Lysine Metabolism Purine Metabolism Proteolysis Metabolism	(63)
Arrhythmogenic Cardiomyopathy (ACM)	Plasma	36 ACM patients, 27 healthy controls	European	Targeted metabolomics with (UHPLC-MS/MS)	Asymmetric dimethylarginine Propionyl C3 carnitine	Alpha-aminoadipic acid, Phosphatidylcholines (PCs and lysoPCs), Tryptophan	Lysine Degradation Beta Oxidation of Fatty Acids Tryptophan Metabolism Arginine and Proline Metabolism	(68)
Dilated Cardiomyopathy (DCM)	Plasma and Urine	273 patients with DCM at varying disease stages (with LVRR, asymptomatic, or symptomatic)	European	Targeted quantitative metabolomics (using internal isotopic standards)	Long chain acylcarnitine's, Sialic acid Glutamic acid, Cystathionine, 3-Methylhistidine, Urine carnosine	Citric acid, 3-hydroxyisovaleric acid, Short and medium-chain acylcarnitine's	Impaired fatty acid, Ketone body metabolism, Mitochondrial dysfunction, Inflammatory pathway, Oxidative stress	(27)
	Serum	106 patients with heart failure due to DCM	European	NMR Spectroscopy	Trimethylamine-N-oxide Creatinine Lactate Succinic acid	Creatine Apolipoprotein-A1,A2 HDL-2,3,4	Energy production pathways Lipid metabolism TCA cycle dysfunction Gut microbiota involvement	(72)
	Plasma	38 patients with DCM, 18 patients with Ischemic CM, 20 healthy controls	Asian (Chinese)	LC–MS/MS untargeted metabolomics	O-Acetylcarnitine, Tyramine-O-sulfate and 3-tyramine, Phosphatidylglycerol (6:0/8:0), Fatty acid esters of hydroxy fatty acids, Phosphatidylcholine (18:0/18:3)	1-Pyrroline-2-carboxylate, Ornithine, Citrulline, Alpha-ketoglutaric acid, Lysophosphatidylinositol (16:0/0:0)	Glycerophospholipid metabolism, Linoleic acid and alpha-linolenic acid metabolism, Arginine and proline metabolism	(73)
Cardiac amyloidosis	Endomyocardial Biopsies	78 patients with cardiac amyloidosis subgroups are: AL (Immunoglobulin Light Chain) Amyloidosis, ATTR (Transthyretin) Amyloidosis, AA (Inflammatory-Related) Amyloidosis	European	retrospectively analyzed from fresh frozen cardiac amyloidosis patients and from 12 biopsies of unused donor heart explants. Nano LC-MS/MS mass spectrometry-based proteomics	Branched-chain amino acids (BCAA) Lipid metabolites (e.g., ceramides) Acylcarnitine's Ketone bodies, ApoE, ApoA-IV and SAP	Glucose metabolism intermediates (e.g., pyruvate and ATP), Taurine, Citrate	Fatty acid oxidation, Glycolysis and gluconeogenesis, TCA cycle, Amino acid metabolism	(75)
	Plasma	27 patients with ATTRV30 M amyloidosis, 26 asymptomatic ATTRV30 M carriers, and 26 controls	European	GC-MS and LC-MS	kynurenine, Xanthine, Niacinamide (S1P)	Amino acids, Glucose, L-Carnitine, Acylcarnitines	Tryptophan metabolism, BCAA metabolism, Purine metabolism, Fatty acid oxidation	(76)
Tachycardia-induced cardiomyopathy response to Atrial fibrillation (AF)	Serum	Cohort of 363 patients. Validation phase (134) Discovery phase (229) patient as following: Healthy control =86 participants, Suspected AF *N* = 30, Cardiogenic ischemic stroke = 32, Other diagnosed AF subtypes =81	Chinese	GC-MS and LC-QTOF-MS	Kynurenine, Xanthine, Niacinamide Sphingosine 1-phosphate (S1P)	Amino acids, Glucose, L-carnitine, Acylcarnitines	Tryptophan metabolism, BCAA metabolism, Purine metabolism, Fatty acid oxidation	(86)
Tachycardia-induced cardiomyopathy response to Heart failure (HF)	Human cardiac tissue	Human cardiac tissue data of 16 HF patients, 19 HF with tachycardia patients and 14 Control Canine model of tachycardia-induced HF, Engineered heart tissue derived from human induced pluripotent stem cells	Caucasian	RNA-seq using canine model of tachycardia-induced HF, Engineered heart tissue derived from human induced pluripotent stem cells	Glucose metabolites (dihydroxyacetone phosphate, pyruvate) Serine Ribulose 5-phosphate Sorbitol	NAD+/NADH ratio	Oxidative phosphorylation TCA cycle Fatty acid oxidation Glycolysis HIF1 signalling Acetylation of sarco/endoplasmic reticulum. Ca2+-ATPase (SERCA2a)	(87)
Takotsubo Cardiomyopathy	Serum	10 Takotsubo patients and 10 control subjects	European	1H-NMR	Ketone bodies (e.g., 3-hydroxybutyrate) 2-hydroxybutyrate Acetyl-L-carnitine Glutamate	Several amino acids including histidine, arginine methionine, Alanine	oxidative stress response pathways lipolysis metabolism, and amino acid metabolism	(88)
Secondary Autoimmune diseases related to Cardiomyopathy	Serum	Meta analysis based-study of patients diagnosed with multiples autoimmune diseases e.g systemic lupus erythematosus (SLE), rheumatoid arthritis (RA), multiple sclerosis (MS), inflammatory bowel disease (IBD)	GWAS database	Two-sample Mendelian randomization approach integrating genetic data and metabolite profiling data	1-Myristoylglycerophosphocholine in RA	Arachidonate (20:4 n6) in IBD, Glycerol in RA, 2-Methoxyacetaminophen sulphate in SLE	Galactose metabolism, Glycine, Serine, and threonine metabolism, Biosynthesis of Unsaturated fatty acids, Glycerophospholipid metabolism, Arachidonic acid pathway	(104)
	Serum	71 Young female adults diagnosed with SLE	Caucasian	Lipidomic analysis targeting total cholesterol and triglyceride	Triglycerides, VLDL-C	HDL-C, LDL-C, APO-A, APO-B	Lipid and phospholipid metabolism	(105)

Overview of metabolic signatures in human models of primary and secondary cardiomyopathy. This table summarizes recent studies that used high-throughput metabolomics techniques to identify the most upregulated and down regulated altered metabolites and dysregulated pathways in cardiomyopathic cohorts across various ethnicities. ATP, adenosine triphosphate; NADH, nicotinamide adenine dinucleotide (reduced form); NADPH, nicotinamide adenine dinucleotide phosphate (reduced form); LVRR, left ventricular reverse remodeling; HDL, high-density lipoprotein; TCA cycle, tricarboxylic acid cycle (also known as the krebs cycle or citric acid cycle); BCAA, branched-chain amino acids; HIF1, hypoxia-inducible factor 1; VLDL-C, very low-density lipoprotein cholesterol; APO-A, apolipoprotein A; APO-B, apolipoprotein B.

### Nutrition deficiency–related cardiomyopathy

Thiamine (vitamin B1) is essential for carbohydrate, lipid, and branched-chain amino acid metabolism, serving as a cofactor for enzymes including pyruvate dehydrogenase and α-ketoglutarate dehydrogenase. Its deficiency disrupts oxidative metabolism, reduces energy production, and promotes pyruvate accumulation, leading to elevated lactic acid levels. This metabolic imbalance impairs cardiac function and contributes to DCM (93). Thiamine supplementation has been shown to improve left ventricular ejection fraction after several weeks of treatment (94). However, its therapeutic efficacy remains limited by the absence of large randomized controlled trials and variability in patient response, necessitating further investigation to establish standardized clinical protocols.

Keshan disease, an endemic CM associated with selenium deficiency, is characterized by myocardial necrosis and fibrosis (95). Selenium, a critical component of selenoproteins such as glutathione peroxidase, mitigates oxidative stress by neutralizing reactive oxygen species (96). Insufficient selenium levels compromise antioxidant defenses, leaving cardiomyocytes exposed to oxidative damage and upregulating pro-apoptotic pathways. Conversely, excess selenium intake has been associated with adverse outcomes, including reduced cardiac output (97, 98). The mechanisms remain unclear, underscoring the need for precise dosing and longitudinal studies.

Hypocalcemia, most often resulting from hypoparathyroidism or vitamin D deficiency, significantly impairs cardiac function by disrupting calcium-dependent myocardial contraction and rhythm regulation (99, 100).

Calcium homeostasis, modulated by parathyroid hormone and 1,25-dihydroxyvitamin D, is essential for coordinating systole and diastole via interactions with the sarcoplasmic reticulum and ryanodine receptors. Hypocalcemia induces metabolic alterations, including increased serum taurine, glutamine, and lipid dysregulation, which compromise cardiac contractility (101). Clinical reports emphasize hypocalcemia's role in congestive heart failure, advocating its inclusion in differential diagnoses (102, 103). Still, management is complicated by heterogeneous etiologies, requiring individualized therapeutic approaches.

### Autoimmune disorders related cardiomyopathy

Notably systemic lupus erythematosus (SLE), rheumatoid arthritis, and dermatomyositis are implicated in secondary CMs through chronic inflammation and immune-driven metabolic dysregulation. In SLE, altered lipid metabolism and xenobiotic pathways enhance oxidative stress and heighten cardiovascular risk (104).

In SLE, reduced levels of 2-methoxy acetaminophen sulfate indicate disrupted xenobiotic metabolism, contributing to oxidative stress and inflammation, both key drivers of CM (104). Zhou et al. further identified dysregulated lipid profiles in young female SLE patients, including reduced Apo A, Apo B, HDL, phosphatidylcholine, and phosphoglyceride, along with elevated triglycerides and VLDL-C (105). These alterations are associated with endothelial dysfunction and increased cardiovascular risk. Persistent inflammation appears to perpetuate these metabolic disturbances, worsening myocardial injury.

In dermatomyositis, elevated betaine levels are associated with impaired amino acid metabolism. While betaine supports methylation and osmoregulation, excess levels can drive the production of TMAO via gut microbiota, an established contributor to atherosclerosis and cardiac dysfunction (106, 107). Monitoring betaine and TMAO levels could help stratify cardiovascular risk in patients with dermatomyositis.

### Toxins related cardiomyopathy

Cardiotoxic agents, including anthracyclines (Doxorubicin), psychotropics, and antiretrovirals, precipitate CM primarily through mitochondrial injury and disruption of lipid metabolism (108, 109). Doxorubicin, for instance, upregulates fatty acid transport genes (FABP4), thereby amplifying cardiotoxicity (110). Balancing therapeutic efficacy against cardiotoxic risks remains a clinical challenge, requiring vigilant monitoring and the development of cardioprotective adjuncts.

Although current evidence supports the role of nutritional deficiencies and cardiotoxic agents in secondary CM, significant gaps remain. Thiamine and selenium supplementation have shown potential benefits, but the absence of extensive, well-controlled studies and variability in methodologies limit definitive conclusions. While hypocalcemia's contribution to cardiac dysfunction is established, management is complicated by heterogeneous etiologies. Autoimmune- and toxin-mediated CM underscore the need for precision medicine, yet current therapies broadly address symptoms rather than underlying metabolic pathways. Future research must focus on longitudinal studies that clarify disease mechanisms and refine therapeutic strategies.

### Challenges and advances in the metabolomics of cardiomyopathy

While metabolomics has emerged as a powerful tool for exploring the biochemical landscape of CM, its application still faces key challenges. High-throughput platforms are susceptible to low-abundance metabolites but often struggle to detect or quantify metabolites present at higher concentrations reliably. Identification is further complicated by variable biochemical properties, such as polarity, which affect detection and reproducibility. Sample integrity is another limitation; exposure to light or fluctuating temperatures can degrade metabolites, compromising consistency.

Moreover, current metabolomic techniques typically rely on relative signal intensities compared to control samples, which reveal trends but lack the absolute quantification needed for clinical decision-making. The complexity of metabolomic data demands a multidisciplinary approach involving clinicians, biochemists, and data scientists to ensure accurate interpretation.

From a technological perspective, traditional one-dimensional chromatography lacks the resolution to separate closely related analytes in complex biological samples. Advanced platforms, such as two-dimensional gas chromatography (2D-GC) and two-dimensional high-performance liquid chromatography (2D-HPLC), offer greater sensitivity, separation, and sample preservation, but at the expense of high costs and technical complexity, which can limit their application in large-scale or translational studies (111–113).

From a translational standpoint, most current CM studies are cross-sectional, rely on small and homogenous cohorts, and use inconsistent sample processing protocols. These issues limit reproducibility and generalizability. Confounding factors, including age, medication use, dietary habits, and lifestyle, further complicate interpretation.

## Conclusion

CM's heterogeneity and lack of specific biomarkers complicate both diagnosis and treatment. Metabolomics offers valuable insights, but its application is limited by technical constraints and insufficient validation. Single-metabolite strategies fail to capture the complexity of metabolic networks, necessitating the integration of multi-omics approaches. This review takes a broader view by comparing metabolic changes across CM subtypes and illustrating their interactions, which provides meaningful insight into disease mechanisms and highlights pathways with diagnostic, prognostic, and therapeutic relevance. This perspective shifts the focus beyond narrow comparisons, making the translational relevance of metabolomics in CM more apparent. We also highlight how advanced techniques, including two-dimensional chromatography and multi-omics integration, can facilitate the development of metabolic biomarker panels with direct clinical applications. Longitudinal studies in diverse cohorts will be crucial for improving diagnostic precision and generalizability. Addressing environmental and comorbid factors is essential, while multidisciplinary collaboration and investment in advanced platforms are vital to advancing metabolomics in precision cardiovascular medicine.
